# The microbial nitrogen cycle

**DOI:** 10.3389/fmicb.2014.00553

**Published:** 2014-10-24

**Authors:** Bess B. Ward, Marlene M. Jensen

**Affiliations:** ^1^Geosciences, Princeton UniversityPrinceton, NJ, USA; ^2^Department of Environmental Engineering, Technical University of DenmarkLyngby, Denmark

**Keywords:** nitrogen cycle, microbial ecology, nitrogen fixation, denitrification, anammox, nitrification

Nitrogen (N) is an essential element in biological systems and one that often limits production in both aquatic and terrestrial systems. Due to its requirement in biological macromolecules, its acquisition and cycling have the potential to structure microbial communities, as well as to control productivity on the ecosystem scale. In addition, its versatile redox chemistry is the basis of complex biogeochemical transformations that control the inventory of fixed (biologically available) N in local environments, on a global scale and over geological time.

Although many of the pathways in the microbial nitrogen cycle were described more than a century ago, additional fundamental pathways have been discovered only recently. These findings imply that we still have much to learn about the microbial nitrogen cycle, the organisms responsible for it and their interactions in natural and human environments. Progress in N cycle research has been facilitated by recent rapid technological advances, especially in genomics and isotopic approaches.

The papers in this issue reflect current research focus on N loss and input processes. The papers are ordered by topic beginning with N fixation, the only biological process that can increase the inventory of fixed N, Knapp (2012) reviewed the literature on the sensitivity of N fixation to dissolved inorganic N and found that neither cultured cyanobacteria nor natural assemblages are completely inhibited by the presence of inorganic N substrates. Knapp was cautious about recent reports of N fixation in subeuphotic mesopelagic waters but concluded that N fixation does occur in the presence of fixed N and in geographic ranges not usually associated with cyanobacteria, which may substantially change our understanding of the global marine N budget. Turk-Kubo et al. (2012) addressed another aspect of the regulation of N fixation and found that different types of N fixers respond differently and variably to Fe or P additions. Both N fixation rates and *nifH* gene expression indicate complex regional and taxonomic sensitivities to micronutrient limitation.

Next we include a series of papers about nitrification, a process which does not directly affect the fixed N inventory, but which links mineralization to the N loss processes by producing oxidized forms of N that can then be used as respiratory substrates. Nitrification has been the subject of increasing research interest since the discovery a decade ago that archaea were involved in ammonium oxidation. A large body of literature has since developed documenting the diversity, abundance and activity of ammonia oxidizing bacteria and archaea (AOB and AOA). For this collection, Casciotti and Buchwald (2012) reviewed knowledge about nitrification gained from the use of N and O isotopes. They found consistent support for the occurrence of nitrification in the euphotic zone, and strong evidence for nitrite reoxidation in suboxic waters. Beman et al. (2012) measured distributions of AOB and AOA in marine sediments and found evidence of their presence as well as active ammonium oxidation in sediments where oxygen was essentially undetectable. They suggest that bioturbation supplies sufficient oxygen intermittently to maintain nitrification even below the typical redox gradient in surficial sediments. Peng et al. (2013) investigated the composition of AOA assemblages in two oxygen minimum zone (OMZ) environments. Although AOA are found in abundance even in waters that contain essentially zero oxygen, active nitrification is not detected there, so Peng et al. (2013) hypothesized that AOA assemblages in oxic waters would differ from those in anoxic waters. Perhaps surprisingly, they found that AOA communities in the OMZ did not differ significantly from those in the overlying surface layer, but they found that biogeography was a significant factor in explaining community composition, as assemblages from the two OMZs (Arabian Sea and Eastern Tropical South Pacific) were significantly different. Bouskill et al. (2012) used trait based modeling to simulate and predict nitrifier community composition and nitrification rates. They found that the relatively simple metabolism of nitrifiers lends itself to such modeling, potentially allowing predictions of the response of nitrification to climate change as reflected in changing environmental parameters such as temperature, pH and substrate availability.

The next topic in the collection deals with the processes by which fixed N is lost from marine ecosystems. Low oxygen environments are of particular interest for nitrogen transformations because they are the sites of fixed N loss via denitrification and anammox. Francis et al. (2013) report on a large sequencing study in sediments of Chesapeake Bay. They found significant geographical patterns in the diversity and composition of denitrifying communities along the estuarine gradient and found that the most abundant types in the environment are only distantly related to anything in culture. Bowles et al. (2012) reported on processes controlling denitrification and the diversity of denitrifying bacteria in the sediments of Guaymas Basin. They found high rates of denitrification associated with *Beggiatoa* mats, but even higher rates in sediments without mats. The presence of sulfide reduced denitrification rates, even though the community contained large numbers of sequences associated with taxa that are capable of linking sulfide oxidation with nitrate reduction. Kirkpatrick et al. (2012) and Fuchsman et al. (2012) report on denitrification and anammox processes and the microbes involved in those transformations in the narrow suboxic zone of the Black Sea water column. Intrusions of oxygen appear to stimulate autotrophic (i.e., sulfide linked) denitrification in the Bosporus plume, while anammox was not detected under these conditions (Fuchsman et al., 2012). In the northeastern gyre of the Black Sea, Kirkpatrick et al. (2012) found that the distribution and level of expression of denitrification genes was more variable than those of anammox genes, although both processes were consistently detected. They suggest that dynamics in the denitrifier population in response to external factors may explain the apparent decoupling between anammox and denitrification in some environments. Sokoll et al. (2012) report on the same N loss processes in the sediments of the Arabian Sea. The two processes showed opposite patterns along the gradient from shallow to deeper sediments, with the relative importance of anammox increasing from 7 to 40% of the fixed N loss at shallow and deep stations, respectively. The sediments have not previously been quantified as a site for fixed N loss in the Arabian Sea but their contribution appears to be significant.

Finally, we include a single paper on the use of nitrogen by the phytoplankton in the surface ocean. Bertrand and Allen (2012) review the evidence for vitamin B deprivation to mediate N limitation in phytoplankton. Nitrogen limitation in phytoplankton may enhance their demand for Vitamins B12 and B1. Interactions between heterotrophic bacteria, cyanobacteria and eukaryotic phytoplankton around the production and demand for vitamins may influence the timing and structure of phytoplankton blooms, including those of harmful algae.

## Conflict of interest statement

The authors declare that the research was conducted in the absence of any commercial or financial relationships that could be construed as a potential conflict of interest.
